# Laparoscopic radical nephrectomy with inferior vena cava thrombectomy: highlight of key surgical steps

**DOI:** 10.1590/S1677-5538.IBJU.2015.0080

**Published:** 2016

**Authors:** A. Sim, T. Todenhöfer, J. Mischinger, O. Fahmy, J. Boettge, S. Rausch, S. Bier, S. Aufderklamm, A. Stenzl, G. Gakis, C. Schwentner

**Affiliations:** 1 Department of Urology, Singapore General Hospital, Singapore; 2 Department of Urology, Eberhard-Karls University Tuebingen, Germany

## Abstract

**Objective:**

Vascular involvement in the form of renal vein (RV) or inferior vena cava (IVC) thrombus can be seen in 4-10% of patients presented with RCC. In patients without presence of metastasis, surgical treatment in the form of radical nephrectomy remains the treatment of choice with 5-year survival rates of 45-70%. Open surgery is still the first treatment option of choice at the moment for RCC patients with IVC thrombus.

**Materials and Methods:**

In our study, we are reporting a case of patient with RCC and level I IVC thrombus treated with laparoscopy. Our patient is a 72 years old man with underlying co-morbidity of hypertension and chronic kidney disease (CKD) presented with right-sided RCC. The CT scan done showed a large right renal upper pole tumor measuring 8.4x5.2cm with level I IVC thrombus (Figure-1). There were no regional lymphadenopathy and the staging scans were negative.

**Results:**

The operative time was 124 minutes and blood loss was minimal. The patient was progressed to diet on POD 1 with bowel movement on POD 2. There was no significant change in the pre and post-operative glomerular filtration rate (GFR). The surgical drain was removed on POD2. The patient was discharged well on POD 5. There were no perioperative complications. The pathology was pT3bN0M0 Fuhrman grade II clear cell RCC.

**Conclusions:**

As a conclusion, laparoscopic radical nephrectomy and IVC thrombectomy is a complex and technically demanding surgery. With advancement of surgical skills as well as technology, more cases of minimally invasive laparoscopic radical nephrectomy and IVC thrombectomy can performed to improve the perioperative outcomes of carefully selected patients in a high volume center.

**Figure 1:**
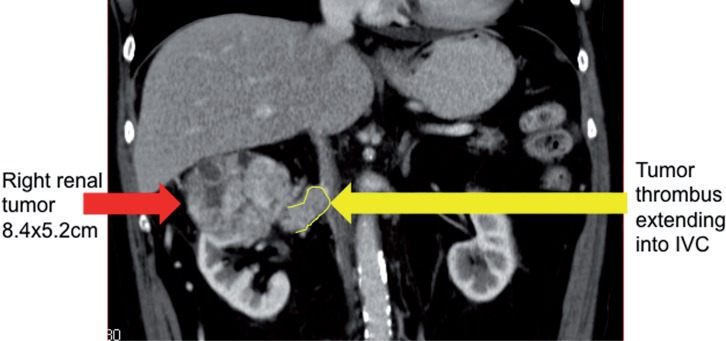
Picture showing pre-operative CT scan showing right renal tumor with thrombus extending into IVC at the level of renal vein.

## ARTICLE INFO

**Available at: www.intbrazjurol.com.br/video-section/sim_856_857/**

**Int Braz J Urol. 2016; 42 (Video #8): 856-7**
